# Postprandial Inflammatory and Metabolic Responses Induced by Authentic Mytilinis Cheese: A Preliminary, Crossover, Nutritional Intervention in Healthy Adults

**DOI:** 10.3390/life13040923

**Published:** 2023-03-31

**Authors:** Olga Papagianni, Angeliki Voutsa, Olga Katira, Panagiota Potsaki, Kalliopi Almpounioti, Konstantina Tzitziri, Dimitrios Skalkos, Antonios E. Koutelidakis

**Affiliations:** 1 Unit of Human Nutrition, Laboratory of Nutrition and Public Health, Department of Food Science and Nutrition, University of the Aegean, 81400 Myrina, Greece; 2 Outpatient Clinic, 81400 Myrina, Greece; 3 Laboratory of Food Chemistry, Department of Chemistry, University of Ioannina, 45110 Ioannina, Greece

**Keywords:** traditional cheese, mediterranean diet, authentic mytilinis cheese, postprandial responses, bioactive components, metabolic biomarkers

## Abstract

Several Mediterranean traditional cheeses may present a beneficial effect on postprandial metabolic and inflammatory modulation due to the presence of bioactive components. The objective of the present preliminary nutritional intervention was the investigation of the postprandial metabolic responses after the intake of traditional Authentic Mytilinis cheese in olive oil with herbs, compared to the corresponding responses after consumption of Italian Parmesan cheese, in healthy participants. A pilot crossover, randomized, single-blinded, intervention clinical trial was conducted in 10 healthy men and women subjects, aged 18–30 years, after random allocation into the control and the intervention groups. The participants received a high-fat carbohydrate meal containing either Authentic Mytilinis cheese (the authentic nonrefrigerated recipe) or Italian Parmesan PDO cheese. After a washout week, the participants consumed the same meals conversely. Differences in the postprandial responses of glucose, triglycerides, uric acid and serum total, HDL and LDL cholesterol levels, as well as of the plasma total antioxidant capacity according to the FRAP method, were determined between groups for fasting, 30 min, 1.5 h, and 3 h after meal intake. The results suggested that meals did not significantly affect postprandial metabolic and inflammatory responses. However, Authentic L Mytilinis cheese resulted in a lower increase of LDL cholesterol (*p* > 0.05) and induced a significant decrease of serum triglycerides (*p* < 0.05) in the last 1.5 h after a meal, compared to Italian Parmesan cheese. Further investigation with large prospective studies is necessary to validate the current findings.

## 1. Introduction

Recent epidemiological evidence has focused on populations’ nutritional and consumer habits [1]. The existing scientific data report that, having as a priority immune system reinforcement and chronic disease prevention, consumers are looking for functional, organic, and nutritionally value-added foods [2]. The Mediterranean diet contains a plethora of traditional, natural functional foods, and their consumption has been incorporated into modern dietary patterns [2]. The great scientific interest around certain traditional functional foods lies in the potential prevention of chronic diseases (e.g., metabolic syndrome, diabetes, cardiovascular diseases, etc.) due to their bioactive compounds (e.g., polyphenols, unsaturated fats, sterols, etc.), which have shown strong anti-inflammatory, antioxidant, and other beneficial activities [3].

Traditional cheeses, as part of the Mediterranean diet, are foods of choice for consumers, and they have been studied for their nutritional value, both in vitro and in vivo [4]. Authentic Mytilinis (AM) is a Greek traditional, hard, yellow cheese in olive oil with herbs, produced based on the traditional recipe and made from Lesvos-breed sheep and goat milk [5,6]. Its unique characteristic is that it is packed with a layer of olive oil, giving it a unique taste and favoring the product’s shelf life [6]. Its frequent consumption may exhibit a protective effect against cardiometabolic diseases by reducing inflammatory biomarkers and atherogenic lipoprotein cholesterol levels, and also by modulating intestinal microflora. The authentic recipe of production includes the placement and preservation of the cheese in olive oil in advance, which yields an innovative, modern, non-refrigerated cheese with unique organoleptic characteristics. It is this “authentic” AM cheese that was used in the current preliminary study.

On the other side, postprandial inflammation and oxidative stress may induce low-grade inflammation, which is directly associated with metabolic diseases, such as atherosclerosis, type 2 diabetes, etc. [7]. Thus, the investigation of the inflammatory and oxidative stress biomarkers in the postprandial state, through nutritional interventions, is a particularly interesting field of study. The acute ingestion of a high-fat and high-carbohydrate meal influences postprandial lipidemic and glycemic metabolism, increasing the magnitude of postprandial inflammation and oxidative stress [7]. Our previous dietary interventions with high-fat and high-carbohydrate meals, including bio-functional foods with bioactive compounds from traditional value-added food sources, suggest a beneficial effect in regulating postprandial inflammation [8,9].

However, the existing evidence regarding the nutritional value of Authentic Mytilinis cheese is still limited. The aim of this preliminary study was the investigation in healthy volunteers of the postprandial lipemic, glycemic, and antioxidant responses to similar high-fat and high-carbohydrate meals containing Authentic Mytilinis (AM) cheese, produced by the Greek cheese company “Thimelis”, compared to Italian Parmesan PDO cheese. Two questions were addressed. (1) What is the acute effect of AM cheese consumption, in the framework of a high-fat and high-carbohydrate meal, on the postprandial biomarkers of lipemia, glycemia, and oxidative stress? (2) Do the postprandial inflammatory and metabolic responses to AM cheese differ, compared to those of Italian Parmesan PDO cheese? The LDL cholesterol and triglycerides levels determined at 3 h after the meal intake were set as the primary endpoint of the present preliminary study.

## 2. Materials and Methods

### 2.1. Study Population

The study protocol was ratified by the University of the Aegean Ethics Committee (No. 7505, 20 October 2021) and was registered at ClinicalTrials.gov database with the Identifier NCT05788887 (public release date: 16 March 2023). The nutritional intervention was carried out according to the guidelines laid down in the Declaration of Helsinki. Participants were recruited from students and staff of the Human Nutrition Unit of the Department of Food Science and Nutrition of the University of the Aegean through an electronic poster and emails in October and November 2022. All volunteers were informed about the ultimate objective of the intervention, the voluntary nature of participation, and the data confidentiality, and they then signed an updated informed consent form.

Inclusion criteria included healthy adults aged between 18 and 65 years and willingness to join and complete the nutritional intervention. Exclusion criteria included the inability or unwillingness to provide informed consent; age <18 and >65 years; three-month nutritional supplement; chronic diseases history; hemoglobin A1c—HbA1c > 5.7%; abnormal Body Mass Index (BMI) (>25 kg/m^2^); abnormal hematological or biochemical profile (total cholesterol > 240 mg/dL, triglycerides > 250 mg/dL, glucose > 100 mg/dL); alcohol overdose (>40 g alcohol/day); heavy smokers (>10 cigarettes/day); concurrent participation in another intervention study; or unconsciousness in previous blood draws. After filling in a short medical history and eating habits questionnaire, anthropometric measurements and initial blood tests were performed in collaboration with external physicians.

Out of the 11 eligible members who provided consent, 1 had an abnormal fasting biochemical profile and dropped out of participation. The final sample size for this interventional pilot study was 10 volunteers. Instructions were provided by staff regarding abstinence from any nutritional supplement or medication during the trial period, as well as from alcohol consumption, 24 h prior to participation, while fasting was requested for at least 12 h before the start of the nutritional intervention.

### 2.2. Meal Challenge Procedure

The meal challenges consisted of cheeses served with 80 g of white bread (two slices of 40 g—produced by a local bakery). The control meal included 100 g of Italian Parmesan PDO cheese (Parmigiano Reggiano—Lovilio, supplied by Lidl Hellas), and the interventional one included the same amount of AM cheese, donated by Greek cheese company E. Thimelis SA (Lesvos, Greece). The total weight of each meal was 180 g. The nutrient composition of the trial meals was calculated with Diet Analysis Plus v. 7.0. (ESHA Research) and is presented in Table 1.

In vitro preliminary determinations of the composition of the Authentic Mytilinis and Italian Parmesan cheeses in Total Antioxidant Capacity (TAC) and total phenolic components was performed for the calculation of the nutritional value of the test meals. Total Antioxidant Capacity (TAC) was measured by the Ferric Reducing Antioxidant power (FRAP) assay, as described by Argyri et al. [10], adding 20 µL of cheese and 80 µL of FRAP reagent in 96-well plate wells and then measuring its absorbance at 595 nm after 30 min in a dark environment. The total phenolic component of each cheese tested was determined by performing the Folin–Ciocalteu method, as described by Ainsworth et al. [11]. Specifically, 50 µL of each sample, 20 µL of 7.5% Na_2_CO_3_ solution, and 20 µL of Folin–Ciocalteu reagent were added in the wells of a 96-well plate, and the absorbance was then measured at 765 nm after 30 min in the dark. The results were quantified using standard curves.

### 2.3. Study Design

**Figure 1:**
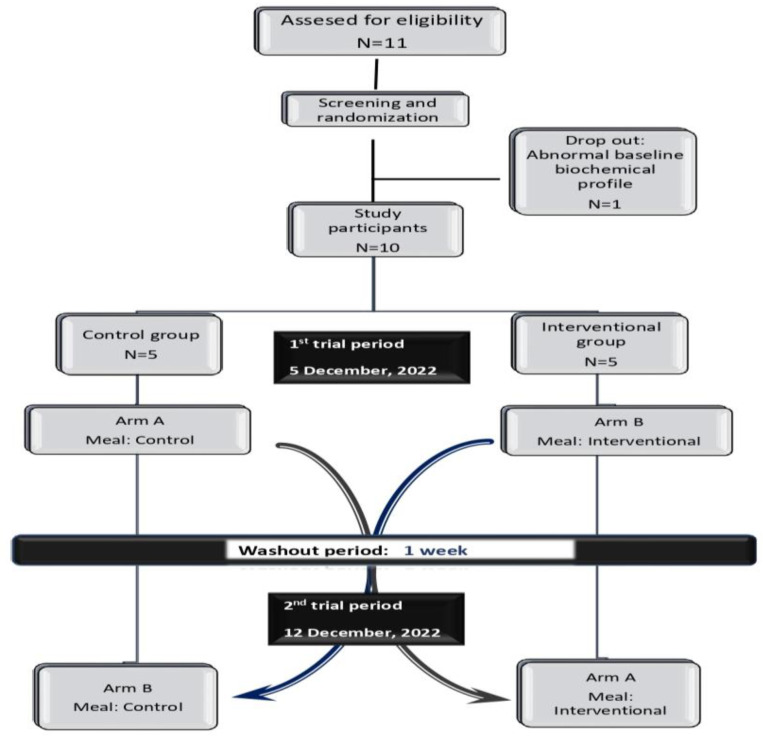
Study design illustration.

The present randomized, crossover, and single-blinded interventional trial was undertaken in the Human Nutrition Unit, Food Science and Nutrition Department, University of the Aegean, on December 2022. Two trial periods were conducted, separated by a washout period of 1 week, during which subjects consumed their usual diet. After screening and baseline data collection, participants were divided into two groups, consisting of five members each, and then assigned at random to the Control (N = 5) or Interventional (N = 5). Volunteers crossed over from one study arm to the other. During each trial period, subjects placed in the control group consumed the meal containing Italian Parmesan PDO cheese, while the interventional group members received the meal containing AM cheese. The study design illustration is presented in Figure 1.

Volunteers visited the Human Nutrition Unit on the trial mornings after fasting 12 h overnight and following the staff instructions. All subjects were requested to fill in a self-administrated 24 h recall questionnaire in order to record the meals consumed in the last 24 h.

Consequently, volunteers were given a meal that weighed 180 g in total, consisting of wheat, white bread, and PDO cheese, and they had access to 250 mL of water. On the trial morning and prior to each visit, the researchers prepared 5 control and 5 interventional meals in subjects’ dishes, which were ID-labelled due to the randomization process. This strategy was followed to avoid any kind of volunteer bias due to knowing the test meal before consumption. All participants were asked to complete the meal within 15 min, under the observation of a laboratory staff member in the framework of this controlled trial. The volunteers were requested to limit physical activity and movement during the trial period and to remain seated as much as possible. Abstinence from smoking and consumption of any other food or drink was requested, although mineral water was provided.

### 2.4. Blood Sampling and Analysis

Baseline fasting blood samples of 10 mL were collected after an overnight fast to determine the concentrations of total, HDL, and LDL cholesterol; triglycerides; glucose; uric acid; and total antioxidant capacity. All participants consumed the test meal within 15 min. At time points 30 min, 1.5 h, and 3 h after the meal completion, blood samples were taken for the determination of blood biomarkers. For serum analyses, blood samples were collected in serum-clot-activator vacuum tubes (Weihei Hongyu Medical Devices Co., Ltd.), while blood samples for plasma analyses were collected in plasma-gel vacuum tubes with Ethylenediaminetetraacetic acid (EDTA, Weihei Hongyu Medical Devices Co., Ltd.). Plasma was immediately separated by centrifugation (20,000× *g* for 10 min, 4 °C) in a tabletop, high-speed, refrigerated centrifuge (Thermo Scientific ST16R, Thermo Fisher Scientific, Waltham, MA, USA) within 30 min of collection and then stored aliquoted at −40 °C in a laboratory freezer (MRC Laboratory Instruments, Holon, Israel) until later analyses. Serum, after 30 min clotting on ice and centrifugation, was isolated, transferred into 1.5 mL aliquots, and immediately frozen at −40 °C until analyzed.

Serum collected at baseline, 30 min, 1.5 h, and 3 h, was used to analyze total, HDL, and LDL cholesterol; glucose; triglycerides; and uric acid. Analysis of the biomarkers mentioned was completed using an automated biochemical analyzer (COBAS c111, Roche, Basel, Switzerland). The plasma Total Antioxidant Capacity (TAC) was determined by the FRAP method, as described by Argyri et al. [8,10].

### 2.5. Data Analysis

Statistical analysis were carried out with SPSS (SPSS V21.0) and Prism 9 (GraphPad Software Inc., San Diego, CA, USA). The sample size was estimated for primary outcomes, with a power analysis for a 2 × 4 factorial, crossover design (2 groups and 4 time points), based on the data of a previous nutrition interventional study with fat-rich meals [3], and via G*Power software version 3.1.9.2 (Universität Düsseldorf).

In order to ensure a sufficient number of participants for 95% confidence to detect a difference between treatments at a two-sided 0.01 significance level, a sample of 10 individuals was needed. Results are expressed as mean ± standard deviation (SD), and at *p* ≤ 0.05, statistical significance was accepted. The Kolmogorov–Smirnov test was applied to check normality. The main participants’ characteristics were analyzed by descriptive statistics. Differences in baseline values between groups for each biomarker tested were analyzed using a paired-sample two-tailed t-test. Repeated ANOVA measures with Geisser–Greenhouse correction were conducted for serum total, HDL, and LDL cholesterol; triglycerides; glucose; uric acid; and plasma Total Antioxidant Capacity (TAC). If a time or group was significant, a multiple-comparison post hoc Bonferroni test was performed for the comparison of concentrations. To check changes in the metabolic biomarkers from baseline to postprandial responses (within-group variation), the Wilcoxon sign-rank test was carried out.

## 3. Results

### 3.1. Preliminary In Vitro Measurements

In vitro preliminary assays showed that AM cheese provided 0.76 mmol FeSO_4_/g of total antioxidant activity and 0.32 mg total phenolics per gram, expressed as gallic acid equivalents, while Italian Parmesan cheese provided 0.96 mmol FeSO_4_/g and 0.33 mg/g GA.

### 3.2. Study Population Characteristics

A total of eleven participants underwent screening. During the initial screening process, one male dropped out due to an excessive baseline biochemical profile. As shown in Figure 1, ten subjects, five men and five women, who met the entry criteria were assigned to one of the two study groups in a random order and in the framework of a crossover design: Control (N = 5) and Interventional (N = 5). After one washout week, each participant crossed over to the remaining group. All subjects completed both study arms, with no attrition. The baseline characteristics of the study population are shown in Table 2.

There were no significant differences in macro- and micronutrients intake, revealed by the dietary analysis of the food frequency questionnaire and the 24 h recall records, which were completed prior to each test day (data not shown). Body Mass Index (BMI) did not significantly differ between genders or among ages. Three volunteers were occasionally light smokers (1–3 cigarettes/day).

### 3.3. Baseline and Postprandial Responses of Test Meals on Metabolic Biomarkers

Table 3 presents the baseline and postprandial changes data for the glycemic and lipidemic profiles and the plasma antioxidant status in the Control and Interventional groups. In addition, Table 3 shows the significance of the group, time effect, and group × time interaction on the postprandial responses to the biomarkers tested. Despite the deviations in the baseline concentration for some biomarkers, due to the randomization process, the importance of our findings is focused on the different postprandial responses of the two test meals.

**Figure 2:**
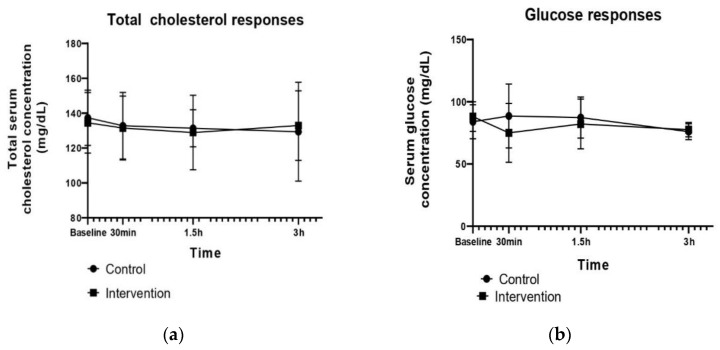

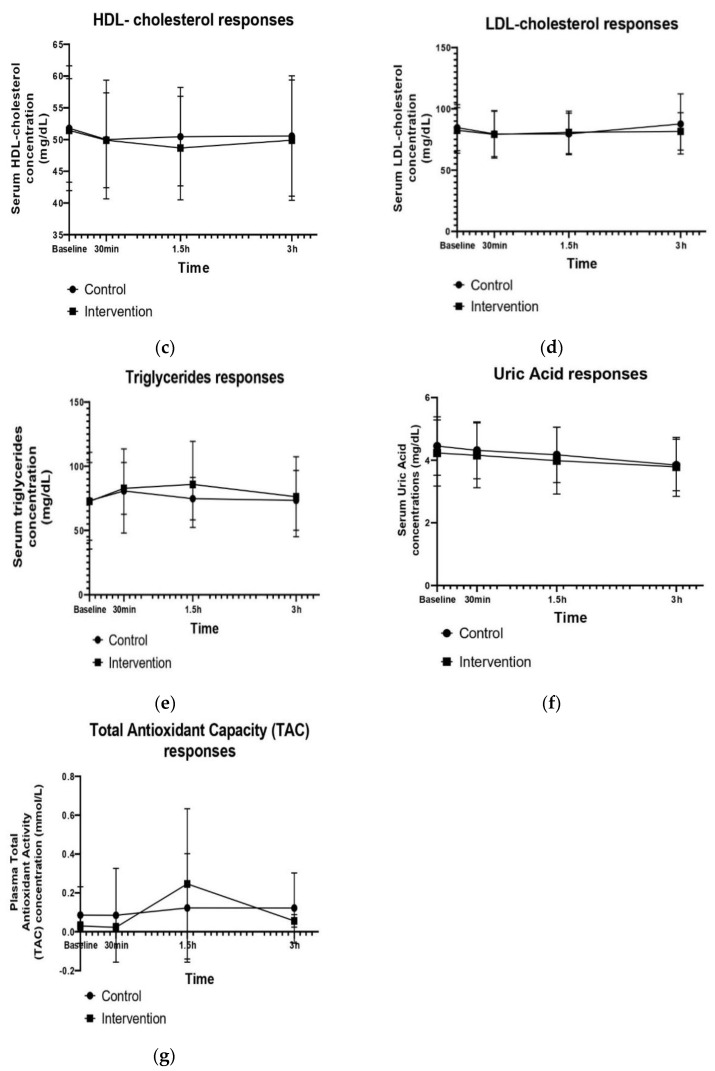
Postprandial responses to test meals of (**a**) Serum total cholesterol, (**b**) Serum glucose, (**c**) Serum HDL cholesterol, (**d**) Serum LDL cholesterol, (**e**) Serum triglycerides, (**f**) Serum uric acid, and (**g**) Plasma Total Antioxidant Capacity (TAC).

Regarding the postprandial responses of the test meals on the serum total cholesterol concentration, there was neither any significant difference between groups at any time point nor any significant group × time interaction (*p* > 0.05) (Table 3, Figure 2a).

The serum glucose concentration was not significantly affected by the meal and the time (*p* > 0.05), which led to no significant group × time interaction (*p* > 0.05), as noted in Table 3. However, as shown in Figure 2b, the interventional meal intake significantly reduced serum glucose levels at 30 min (*p* = 0.041, MD_Baseline-30min_ = 8.9), while no significant changes were observed 30 min after the control meal intake (*p* > 0.05, MD_Baseline-30min_ = −3.6). Analysis showed that the postprandial glucose values decreased 1.5 h after both meals, but only the control meal led to significant changes (*p* = 0.019).

Time significantly affected HDL cholesterol values (significant time effect, *p* = 0.05) from baseline to 1.5 h, but no group effect or group × time interaction was found (*p* > 0.05) (Table 3, Figure 2c).

As shown in Figure 2d, the resulting LDL cholesterol values were significantly reduced at 30 min after consumption of the control (*p* = 0.006, MD_Baseline-30min_ = 4.64) and interventional (*p* = 0.003, MD_Baseline-30min_ = 3.3) meals, but a significant increase was observed in the LDL cholesterol concentration at the last 1.5 h (*p* = 0.008, MD_1_._5h–3h_ = −7.2) after the control meal intake, while in the interventional group, in the same period, a milder, non-significant increase was found (*p* > 0.05, MD_1_._5h–3h_ = −2.2). Statistical analysis indicated a significant time effect (*p* = 0.0004, Table 3) from baseline to 30 min and from 30 min to 3 h (MD_Baseline-30min_ = 3.967 and MD_30min–3h_ = 6.076). Nevertheless, no significant differences between groups (group effect, *p* > 0.05) and no group × time interaction (*p* > 0.05) were observed (Table 3).

Figure 2e illustrates the postprandial changes of serum triacyloglycerols after the consumption of high-fat and high-carbohydrate meals containing Ladotyri Mytilinis or Italian Parmesan cheese. As the results show in Table 3, the postprandial concentration of triglycerides was not affected by the group or time (group and time effect, *p* > 0.05), while no significant group × time interaction was found (*p* > 0.05). Further analysis indicated that serum triglycerides peaked at 1.5 h and significantly decreased from 1.5 h to 3 h (*p* = 0.008, MD_1_._5h–3h_ = 9.7) after consumption of the interventional meal containing Authentic Mytilinis cheese. In the same time period, no significant response was noted after the control meal consumption (*p* > 0.05). The triglycerides concentration peaked at 30 min after Italian Parmesan administration, followed by a mild decrease until 3 h.

The postprandial responses of test meals in the serum uric acid values are presented in Figure 2f. A significant decrease was found (time effect, *p* = 0.0004, Table 3) from the baseline to any postprandial time point, until 3 h (MD_Baseline-30min_ = 0.105, MD_Baseline-1_._5h_ = 0.263 and MD_Baseline-3h_ = 0.524), after the intake of both meals. However, no significant group effect and no group × time interaction were detected for this metabolic biomarker (*p* > 0.05,Table 3).

The postprandial responses of plasma Total Antioxidant Capacity (TAC) seem to not differ between the study groups (group effect, *p* > 0.05) and the time points (time effect, *p* > 0.05), and no significant group × time interaction was observed (*p* > 0.05) (Table 3). As illustrated in Figure 2g, a different response of plasma TAC was observed for meal challenges. Specifically, an increase in plasma TAC values was found from 30 min to 1.5 h (MD_30min–1_._5h_ = −0.155), followed by a decrease at the last 1.5 h (MD_1_._5h–3h_ = 0.122) after consumption of the meal containing Authentic Mytilinis cheese. On the other hand, the intake of the control meal containing Italian Parmesan cheese led to slightly elevated plasma TAC levels from 30 min to 1.5 h (MD_30min–1_._5h_ = −0.056, 11.8%), followed by a slight decrease until 3 h (MD_1_._5h–3h_ = 0.05). Despite this variation in the plasma TAC response, the aforementioned changes failed to reach statistical significance.

## 4. Discussion

In this preliminary study, the impact of Authentic Mytilinis (AM) and Italian Parmesan cheese consumption, in the framework of a high-fat carbohydrate meal, on postprandial lipemia, glycemia, and oxidative stress biomarkers was compared in healthy volunteers.

The degree of postprandial inflammation and oxidative stress induced by a fatty, high-carbohydrate meal depends on several nutrient-dependent factors. The total energy intake, provided carbohydrates, and meal glycemic index, as well as the concentration and composition of lipids, can lead to a variable postprandial lipid, glycemic, and antioxidant response [7]. In addition, the protein content, food texture, composition in micronutrients, and bacterial content may affect the kinetics of food decomposition and the bioavailability of dietary lipids [12]. Cheeses show significant variations in terms of their nutrient content, as well as in the cheese matrix. The cheese matrix, depending on the hardness and consistency, may delay the digestion of dairy fats and reduce the postprandial lipid response [12].

There is evidence that meals with a similar amount of fat from different dairy products induce a different impact on postprandial lipemia in healthy subjects [12,13], and the fatty acids profile of cheese may be predictive for the cholesterol effect [14]. Further, findings from long-term studies suggest that cheese consumption, in contrast with other fat dairies, such as butter, results in a lower cholesterol level [14]. In this pilot acute and interventional study, no significant effect of the meal or time on postprandial cholesterol levels was observed. The study design, the amount of cheese ingested, and the metabolic capacity of each subject, as well the short duration of the postprandial measurements, may affect our findings. Despite the fact that existing scientific data suggest that dairy fat raises HDL cholesterol [15], Hjerpsted et al. reported that cheese resulted in a decrease of HDL cholesterol, compared to butter [14]. Looking at previous studies, it was observed that the consumption of similar meals seems to affect the levels of HDL cholesterol over a longer timeframe (0–6 h) [13].

Although no significant differences were observed between two groups, the glucose time to peak was delayed by the intervention meal (t = 1.5 h) in comparison to the control (t = 30 min). Although the total amount of carbohydrates in a meal is the most important component that primarily influences the postprandial glycemic response, it is well established that the fats and proteins in cheese delay gastric emptying, and this in turn delays the rise in postprandial glycemia. In addition, proteins may increase insulin secretion and thus decrease the glycemic response [16,17]. Several studies indicated that phenolic compounds from olive oil have an impact on postprandial glucose. Klisović et al. suggested that when a hard cheese was saved in extra virgin olive oil for two months, the total phenolic components increased significantly [18]. Thus, the decrease of the glucose concentration 30 min after the Authentic Mytilinis cheese consumption was possibly attributable to the action of the polyphenols derived from the cheese. In the present pilot clinical study, glucose levels returned to the fasting levels after 3 h, in accordance with other studies. However, it has been reported that carbohydrate meals high in fats and protein may increase postprandial glucose even after 3 to 5 h post-meal [13].

In the present preliminary study, LDL cholesterol levels followed a downward trend at the first 30 min after the intake of both meals containing either Authentic Mytilinis (AM) or Italian Parmesan cheese. However, in the last 1.5 h, a greater increase in the concentration of LDL cholesterol was observed in the group that received the Italian Parmesan meal, compared to the group that consumed the AM meal. The total energy and fat, as well as the protein and phenolic contents, was similar in the tested cheeses and meals, but the antioxidant activity of the cheeses differed, with Italian Parmesan having the highest total antioxidant capacity.

These findings are in accordance with other interventional studies examining the postprandial response of LDL cholesterol to meals that contain cheese [19]. The decrease in LDL cholesterol values could be attributed to a cheese matrix effect, possibly due to the entrapment of the fat globules of the examined cheeses within the casein matrix formed by aggregated micelles [14]. There are indications that the lactic acid bacteria (mainly Lactococcus), used during the production process of the cheeses under study, exert a hypocholesterolemic effect. It is possible that the fermentation process, to which both cheeses are subject [20,21], plays a role in reducing LDL cholesterol in the postprandial state [14]. A possible mechanism that has been proposed is that, under the action of lactic acid bacteria, short-chain fatty acids are produced from unabsorbed carbohydrates, which may alter cholesterol synthesis. In addition, bile acid sequestration by these bacteria is likely, leading to reduced bile acid recycling in the enterohepatic circulation [14]. Moreover, calcium contained in dairy products has been found to play a role in reducing fat absorption, as well as the rate of increase in total and LDL cholesterol, caused by long-term intake of saturated fat, leading to postprandial lipemia reduction [13].

It is known that Authentic Mytilinis cheese, as indicated by its name, is preserved in olive oil [20]. The presence of olive oil during the production process of Authentic Mytilinis cheese seems to enhance the cardioprotective effect of its consumption [6]. The lower-grade increase of the LDL cholesterol concentration after receiving AM, compared to Italian Parmesan, may reflect the bioactivity of the olive oil contained in Authentic Mytilinis cheese. Previously, Pastor et al. suggested that the intake of oleic acid, provided by olive oil, is associated with an improved or unchanged lipid profile, mainly due to a reduction in total and LDL cholesterol [22].

The second finding of this pilot nutritional intervention was the significant reduction of the triglycerides concentration 3 h after the consumption of AM, whereas a milder decrease was found at the same time after the intake of Italian Parmesan. Nevertheless, examining the overall response of serum triglycerides, it was observed that the triglycerides levels did not significantly differ between both groups, compared to the respective initial concentration, before the administration of the test meals. Several studies indicate an inverse correlation of the dietary lipid droplet size, in the cheese matrix, and the postprandial triglyceride response [12]. It is worth mentioning that the small difference found in the postprandial response of triacylglycerols may be attributed to the presence of oleic acid in the matrix of AM; there is evidence that oleic acid may increase the size of triacylglycerol-rich postprandial lipoproteins. Ohlsson et al. reported that replacing SFAs with MUFAs in a meal may induce the formation of larger postprandial chylomicrons [23].

The addition of antioxidant components to high-fat and high-carbohydrate meals may have a protective effect against postprandial oxidative stress and endothelial dysfunction [24]. In the present pilot clinical study, although our preliminary in vitro experiments showed higher antioxidant activity in Italian Parmesan than in Authentic Mytilinis, small differences in the response of the volunteers’ plasma antioxidant capacity were found. The total plasma antioxidant capacity of subjects who received AM peaked at 1.5 h after the meal intake, presenting at the same time a higher concentration of antioxidants than the volunteers who consumed Italian Parmesan, but this beneficial effect weakened until 3 h. This activity could be attributed to corresponding possible changes in the values of blood uric acid, as it is an endogenous antioxidant and could enhance the overall antioxidant capacity, but the uric acid response curves do not justify such an effect. Possibly, the concentration of antioxidants provided by the olive oil in AM was not enough to maintain the plasma antioxidant activity. Even so, it has been reported by Murray et al. that there is a lack of sufficient postprandial time points for the determination of the incremental changes in biomarkers of oxidative stress and antioxidant status over the postprandial period [25]. There is also debate regarding the measurement of antioxidant status general indicators, and the establishment of more appropriate antioxidant biomarkers is needed [25].

Some limitations of the present preliminary study should be underlined. This was a pilot nutritional intervention, and even though the adequacy of the participant sample size was statistically calculated, the small number of participants may have influenced the lack of statistical significance for the biomarkers tested. Therefore, larger studies should be performed using the data of this preliminary study, and further studies with a higher number of participants, both healthy and with a high metabolic risk, should be conducted. Furthermore, no standardized meals were provided for the participants in the evening prior to the test days, which might have affected their fasting values. Another limitation of the study is that extra analyses could have been carried out to evaluate inflammatory biomarkers and determine the antioxidant capacity. The establishment of new metabolic biomarkers to investigate postprandial inflammation and oxidative stress after the consumption of a high-fat and -carbohydrate meal, including a Greek and an Italian cheese, is a primary future goal. In addition, the duration of this acute study was 3 h, an amount of time that is not adequate for the full metabolism of HDL and LDL cholesterol. It would be interesting to add new time points for blood sampling in order to examine the effect of consuming Ladotyri and Parmesan cheeses up to 6 h after receiving the meal and to draw more accurate conclusions regarding the metabolism of their macronutrients and micronutrients.

## 5. Conclusions

The findings of the present pilot study indicate that the supplementation of a high-carbohydrate meal with fat from an Italian PDO or Greek cheese with similar organoleptic characteristics, as well as fat and protein content, may induce a slight but non-significant difference in the postprandial response of metabolic biomarkers of lipemia, glycemia, and oxidative stress. The potential metabolic pathways and the clinical impact of these findings require additional studies in order to draw safer conclusions.

## Figures and Tables

**Table 1:** Dietary composition of the tested meals.

Nutrients	Control	Interventional
Total weight (g)	180	180
Energy (kcal)	615.6	596.6
Fat, total (g)	32.57	35.87
Saturated fat (g)	20.13	19.33
Carbohydrates (g)	39.36	40.06
Protein (g)	39.94	28.24
Salt	2.64	5.54
TAC *	0.96	0.76
TPC *	0.33	0.32

* TAC: Total antioxidant content (mmol Fe_2_^+^/g), TPC: Total phenolic content (mg gallic acid/g).

**Table 2:** Study participants baseline characteristics.

Volunteers Baseline Characteristics	
Volunteers (number)	10
Men (number)	5
Women (number)	5
Dietary supplementation (number of participants)	0
Physical activity medium or high (number of participants)	5
Age (years)	23 ± 3.01
Weight (kg)	72.18 ± 10.3
Height (cm)	170.2 ± 7.2
Body Mass Index	24.86 ± 3.01

Values for age, weight, height, and Body Mass Index represent mean ± SD.

**Table 3:** Baseline clinical characteristics and postprandial responses of serum total, HDL, and LDL cholesterol; glucose; triglycerides; uric acid; and plasma Total Antioxidant Capacity (TAC) following a high-carbohydrate and high-fat meal containing an Italian Parmesan or an Authentic Mytilinis cheese.

	Total Cholesterol at Baseline (mg/dL)	Δ30 min	Δ1.5 h	Δ3 h	*p*-Value Group Effect	*p*-Value Time Effect	*p*-Value Group × Time Interaction
Control	137.33 ± 15.82	−4.56 ± 3.2	−1.44 ± 1	−2 ± 1.4	0.871	0.322	0.621
Intervention	134.44 ± 17.38	−3 ± 2.1	−2.56 ± 1.8	4 ± 2.8			
	Glucose at baseline (mg/dL)	Δ30 min	Δ1.5 h	Δ3 h	*p*-Value Group Effect	*p*-Value Time Effect	*p*-Value Group × Time Interaction
Control	83.92 ± 13.64	3.6 ± 2.5	−2.3 ± 1.6	−10.2 ± 7.2	0.817	0.232	0.516
Intervention	88.07 ± 11.9	−8.9 ± 6.3	1.7 ± 1.2	−5.9 ± 4.2			
	HDL cholesterol at baseline (mg/dL)	Δ30 min	Δ1.5 h	Δ3 h	*p*-Value Group Effect	*p*-Value Time Effect	*p*-Value Group × Time Interaction
Control	51.77 ± 9.83	−1.78 ± 1.3	0.44 ± 0.3	0.11 ± 0.1	0.527	0.050	0.682
Intervention	51.44 ± 8.15	−1.56 ± 1.1	−1.22 ± 0.9	1.22 ± 0.9			
	LDL cholesterol at baseline (mg/dL)	Δ30 min	Δ1.5 h	Δ3 h	*p*-Value Group Effect	*p*-Value Time Effect	*p*-Value Group × Time Interaction
Control	82.45 ± 18.58	−4.64 ± 3.3	0.34 ± 0.2	7.72 ± 5.5	0.335	0.0004	0.454
Intervention	82.46 ± 18.58	−3.3 ± 2.3	1.89 ± 1.3	2.21 ± 1.6			
	Triglycerides at baseline (mg/dL)	Δ30 min	Δ1.5 h	Δ3 h	*p*-Value Group Effect	*p*-Value Time Effect	*p*-Value Group × Time Interaction
Control	73.22 ± 37.7	7.6 ± 5.3	−6 ± 4.2	−1.3 ± 0.9	0.759	0.220	0.536
Intervention	72.66 ± 30.38	10.1 ± 7.1	3.1 ± 2.2	−9.7 ± 6.8			
	Uric acid at baseline (mg/dL)	Δ30 min	Δ1.5 h	Δ3 h	*p*-Value Group Effect	*p*-Value Time Effect	*p*-Value Group × Time Interaction
Control	4.45 ± 0.93	−0.14 ± 0.1	−0.15 ± 0.1	0.32 ± 0.2	0.441	0.0001	0.348
Intervention	4.22 ± 1.05	−0.07 ± 0.05	−0.17 ± 0.02	−0.2 ± 0.04			
	Total Plasma Antioxidant Capacity (TAC) (mmol/L)	Δ30 min	Δ1.5 h	Δ3 h	*p*-Value Group Effect	*p*-Value Time Effect	*p*-Value Group × Time Interaction
Control	0.086 ± 0.14	0.012 ± 0.008	0.056 ± 0.04	−0.05 ± 0.03	0.378	0.256	0.720
Intervention	0.029 ± 0.021	−0.01 ± 0.007	0.155 ± 0.08	−0.12 ± 0.08			

## Data Availability

The data presented in this study are available within this article.
